# Seroprevalence of HIV infection among the patients attending various emergency departments in a tertiary care hospital

**DOI:** 10.4103/2589-0557.68997

**Published:** 2010

**Authors:** Pushpa Devi, Usha Arora, Shalini Yadav, Sita Malhotra

**Affiliations:** Department of Microbiology, Government Medical College & Hospital, Amritsar, Punjab

**Keywords:** Emergency department, human immunodeficiency virus, rapid test, universal work precautions

## Abstract

Emergency departments (EDs) receive patients from every background, socioeconomic group and health status. Hence, EDs can play a critical role in offering human immunodeficiency virus (HIV) testing and help in the national strategy of early HIV detection. The present study was conducted on 400 patients attending various EDs after taking Institutional Review Board approval. They were screened for HIV antibodies by three rapid/simple assay tests having different principles/antigens as per the NACO guidelines. Twenty-three (5.75%) of the 400 patients were HIV reactive. Fifteen (65.22%) of the 23 HIV-reactive patients were unaware of their reactive status. Majority of the HIV-reactive (65.22%) patients were from the Medicine emergency followed by Orthopaedics and Surgery (13.04%). Seven (30.43%) had history of fever of more than 1 month duration. Eight (34.78%) of them were later on clinically diagnosed as having various opportunistic infections. Thus, the study emphasizes the need for expansion of routine voluntary HIV counseling and testing to all the patients who come to the ED and practicing universal work precautions by health care workers.

## INTRODUCTION

The emergency department (ED) is an ideal place for public health interventions and provides ready access to the health care system, offering a great opportunity for human immunodeficiency virus (HIV) testing and counselling.

EDs receive patients from every background, socioeconomic group and health status. Hence, EDs are a key component of the health care safety net. Patients not linked with health services are particularly likely to seek care in an ED when medical concern arises.[1] However, it has been seen that HIV infection is diagnosed most of the times with one or the other opportunistic infection (OI), particularly in developing countries. At the time of diagnosis, most of the patients have advanced disease and are at a higher risk of OIs and death. Emergencies in HIV-infected patients can occur at any stage of the disease. EDs can play a critical role in offering HIV testing and help in the national strategy of early HIV detection. Early detection will allow the infected individuals to take full advantage of Anti retroviral therapy (ARTs) and preventive medicines for opportunistic infections.[2] Emergency medical personnel must assiduously guard against accidental needle stick injury by practicing universal work precautions (UWPs) strictly as they present the greatest risk for health care work-related HIV infection.[3]

## MATERIALS AND METHODS

The present study was conducted on 400 patients from various EDs of Medicine, Surgery, Obst and Gynaecology and Orthopaedics attached to the Government Medical College, Amritsar, for HIV antibodies after taking approval from the Institutional Review Board. Detailed history was taken. Pre-test counseling of the patients was performed and their informed consent was taken before blood sample collection. HIV antibodies were detected by various rapid/simple assay tests using different antigens and principles as per the NACO guidelines provided at ICTC, Government Medical College, Amritsar.

Patient’s serum was first tested using COMB AIDS-RS (HIV 1and 2 Immunodot test kit) from Span Diagnostics Ltd. (Town center, 303 Andheri Kurla Road Marol Andheri-East, Mumbai India) If found to be reactive for HIV antibodies, this was confirmed using Retroqic HIV (Rapid Immunoconcentration HIV I and II Antibodies) from Qualpro Diagnostics (1^st^ Floor plot 88-89, Phase 2C Verna Industrial Estate, Goa, India.) and HIV Tridot for HIV I/II antibodies from J. Mitra and C. Pvt. Ltd. (A180/181 Okhla Industrial are Phase-1, New Delhi, India.) The results were tabulated and analyzed using the chi-square test and *P*-value <0.05 was taken as statistically significant. Post-test counseling was carried out for those who were found to be HIV reactive and were referred to the ART Center attached to the Government Medical College, Amritsar, for treatment.

## RESULTS

Of the 400 patients in the study group, 23 (5.75%) were found to be HIV reactive, as shown in Table 1. Fifteen (65%) of the 23 HIV-reactive patients were not aware of their HIV infection.

**Table 1:** Demographic profi le of the study population (n = 400)

	HIV positive	HIV negative	Total	% age of HIV positive
Sex				
Male	15	194	209	7.17
Female	8	183	191	4.18
Total	23	377	400	5.75
Agegroup				
<20	2	45	47	4.25
21–30	8	127	135	5.92
31–40	11	85	96	9.57
41–50	2	51	53	7.27
>50	0	69	69	-
Occupation				
Government employee	2	29	31	6.45
Farmer	4	73	77	5.19
Housewife	7	147	154	4.54
Laborer	4	67	72	5.97
Shopkeeper	0	40	40	-
Student	1	17	18	5.8
Truck driver	5	3	8	62.5
Maritalstatus				
Married	20	286	306	6.53
Unmarried	3	91	94	3.19

HIV seroreactivity with respect to sex, age, occupation and marital status of the 400 patients under study group is shown in Table 1. Twenty-one (91.30%) of the 23 HIV-reactive patients were in the age group ≤40 years, while only two (8.69%) were >40 years old. The difference in the incidence of HIV among the different age groups is statistically significant (*P* = 0.0175).

HIV infection among the people of different occupations is shown in Table 1. Of eight truck drivers, five (62.5%) were HIV positive (*P* < 0.001) and thus the difference is statistically significant.

Various risk factors associated with the HIV-reactive patient are shown in Table 2. ED-wise distribution of 400 cases and the percentage of HIV seropositivity among the patients of different departments is shown in Table 3.

**Table 2:** Risk factors present in HIV-reactive patients

Risk factors	No. of cases	% age
Multiple sex partners	14	60.86
Intravenous drug users (IVDUs)	7	30.43
MSP + IVDU	1	4.34
Contaminated needle*	1	4.34

* probable cause – as history of multiple injections given by quacks for treatment of pyrexia of unknown origin

**Table 3:** HIV seropositivity among 400 patients attending various emergency departments

Department	No. of cases	No. of HIVpositive cases	% age of HIVpositive cases
Medicine	152	15	9.8
Surgery	86	3	3.5
Orthopaedics	82	3	3.6
Gynaecology and obst.	80	2	2.5

Various emergencies present in HIV-reactive patients in various EDs are shown in Table 4.

**Table 4:** Various emergencies present in HIVpositive patients (n = 23)

Type of emergency	Total no. of patients	Percentage
Fever >1 month duration	07.0	30.43
Gastroentertis >1 month duration	01.0	04.35
Chronic cough	02.0	08.70
Diffi culty in swallowing and ulcers in mouth	03.0	13.04
Neurological disorders	02.0	08.70
Various fractures	03.0	13.04
Various surgical problems	03.0	13.04
Pregnancy with labor pains	02.0	08.70

## DISCUSSION

In serological surveys conducted by the Centre for Disease Control (CDC), Atlanta, 0.2-8.9% of the patients seeking emergency care and 0.1-7.8% of the patients receiving acute care were found to be HIV antibody positive. Sixty-three to 65% of these patients were unaware of their HIV status.[4] In India, Teja *et al*, have reported an incidence of 2.95% in EDs.[2] Many other studies have shown that ED can play a major role in the diagnosis of HIV infection.[5]

In 2001, the CDC had issued guidelines giving explicit emphasis to the role of emergency physicians, owing to the realization that the ED represents the only source of medical care for many patients and often serves as the primary site for routine health care to communities at risk for HIV.[6] A high number of patients (65.22%) in our study were unaware of their infection, which might be because of the reason that the people in routine do not need to go for HIV testing on their own without the onset of serious symptoms. It is evident that ED can play a critical role in offering HIV testing and help in the National Strategy of early detection and further prevention programmes.[7]

As the HIV seropositivity rate in emergency patients is alarmingly high in the present study, there is the need of early diagnosis of primary HIV infection, which represents an important opportunity to prevent transmission to others as, in the early stages of the disease, patients have high levels of viremia, which, coupled with a lack of awareness, can lead to transmission to family and others.[2] Early detection of primary HIV infection will also help the HIV-reactive patients in starting their treatment in time. This will also help health care personnel to understand the importance of practicing UWPs while rendering care to patients to prevent transmission of HIV infection, particularly inexperienced junior residents practicing emergency medicine in an environment that is often frenetic.

HIV testing should be integrated with screening for other infections such as viral hepatitis, sexually transmitted diseases and tuberculosis. Because populations disproportionately affected by HIV are also disproportionately affected by these infections, integrating these services can significantly improve health care services.

Thus, the study emphasizes the importance of offering rapid HIV testing to all patients who present in the ED with or without symptoms. Routine testing might increase the linkage of HIV-positive persons to health and prevention services earlier in the course of infection, which might result in improved long-term prognosis and reduced HIV transmission.
